# Supplementary material for: Highly structured homolog pairing reflects functional organization of the Drosophila genome
Source: Nat Commun. 2019 Oct 3;10:4485. doi: 10.1038/s41467-019-12208-3 (PMC6776532; doi:10.1038/s41467-019-12208-3)
Supplement: Supplementary file 1 — Supplementary Information [file 41467_2019_12208_MOESM1_ESM.pdf]

Supplementary Information

Highly structured homolog pairing reflects functional level of organization of the *Drosophila* genome

AlHaj Abed *et al*.

Highly structured homolog pairing reflects functional level of organization of the *Drosophila* genome

Jumana AlHaj Abed*, Jelena Erceg*, Anton Goloborodko*, Son C. Nguyen, Ruth B. McCole, Wren Saylor, Geoffrey Fudenberg, Bryan R. Lajoie, Job Dekker, Leonid A. Mirny†, Ting (C.-ting) Wu†

*These authors contributed equally to this work.

† Correspondence to: leonid@mit.edu (L.A.M.); twu@genetics.med.harvard.edu (C.-t.W.)

Supplementary figures and legends

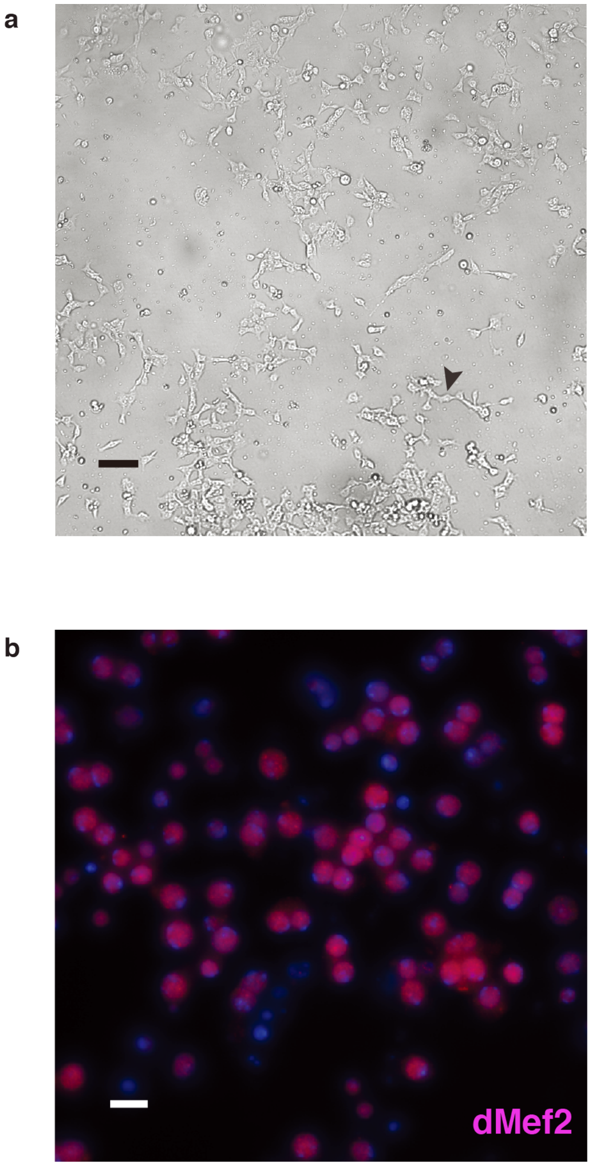

Supplementary Figure 1. PnM cell line morphology and cell-type characterization. a, PnM cell culture consists of adherent, bipolar shaped cells (arrow head). The culture is about 70% confluent and is ready to passage. Bar = 40 µm. **b,** PnM cells stained for DAPI and the mesodermal cell marker dMef2, suggested they are predominantly of mesodermal origin. Bar = 10 µm.

a

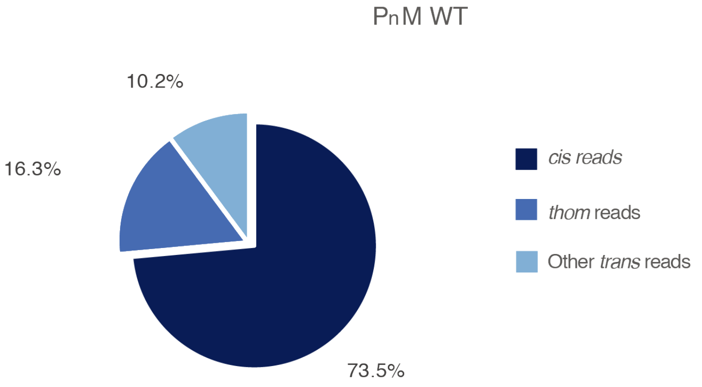

b

	PnM	2-4h Embryos
**Total mappable reads (1e6)**	75.38	49.05
***cis* reads (1e6)**	55.44	46.21
**% *cis* reads of total**	73.55%	94.20%
***trans* reads (*thom* & *thet*) (1e6)**	19.95	2.84
**% *trans* reads (*thom* & *thet*) of total**	26.47%	5.79%
**% *thom* reads**	16.29%	2.10%
**% *thet* reads**	10.16%	3.68%
cis: trans (thom* & *thet)	2.78	16.25
**(PnM:Embryo) *cis***	---	1.20
**(PnM:Embryo) *trans***	---	7.02

c D

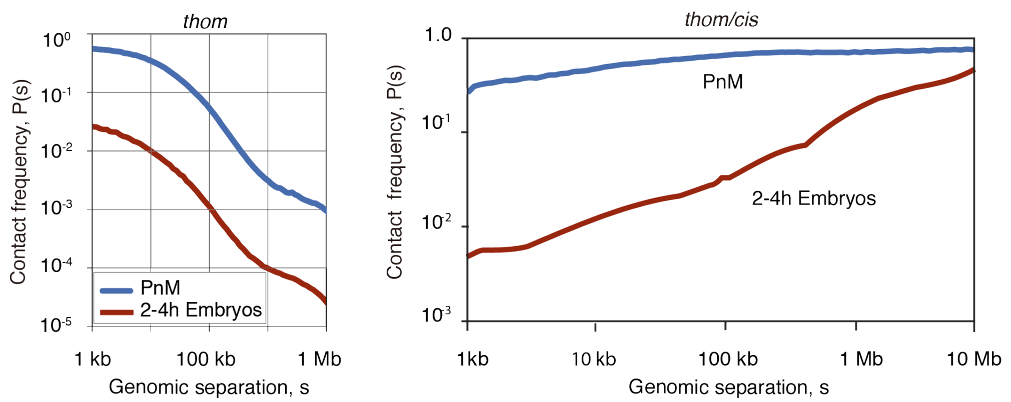

Supplementary Figure 2. *Trans* reads are highly abundant in PnM cells. a, Pie chart comparing percentage of non-duplicated *cis* and *trans* reads recovered after haplotype-resolved mapping to hybrid genome in PnM, where *cis* interactions refer to read pairs with a SNV on each side mapping to the same homolog and *trans* interactions refer to read pair with a SNV on each

side mapping to a different homolog or chromosome. Data from PnM untreated cells showing PnM total *trans*-reads are almost a quarter of total reads. **b,** Comparison of mappable, non-duplicated reads in PnM and 2-4 h embryos[1] shows that the percent of *trans*-homolog (*thom)* mappable reads in PnM (16.29%) are ~8-fold those of embryos (2.10%). **c,** *Thom* signature in PnM cells is greater than *thom* in embryos at all linear separations, s, in kilobases (kb). **d,** *Thom*-to-*cis* contact frequencies in 2-4 h embryos and cells. Around 100 kb separation in PnM cells, *thom* interaction frequency is almost equal to *cis* interaction frequency. This contrasts to the pairing signature that is observed in 2-4 h old embryos.

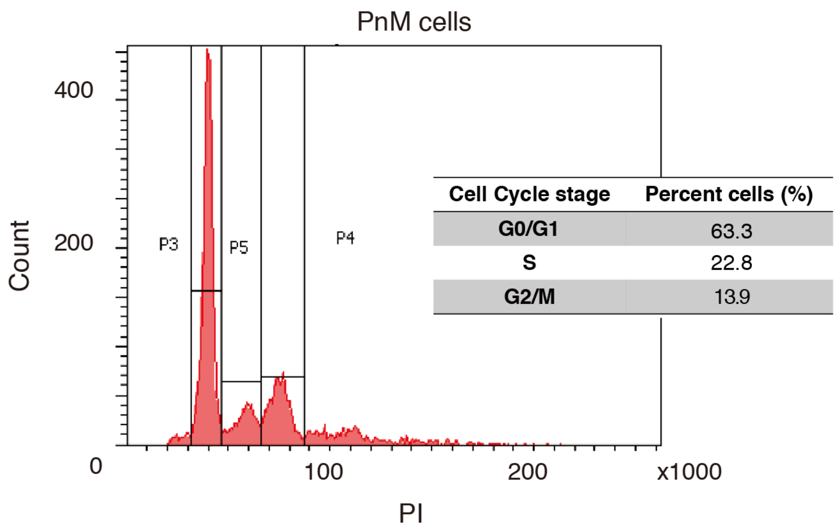

Supplementary Figure 3. Cell cycle analysis of PnM cells. Staining of cells with propidium Iodide at 70% confluency showed that they are predominantly in G1.

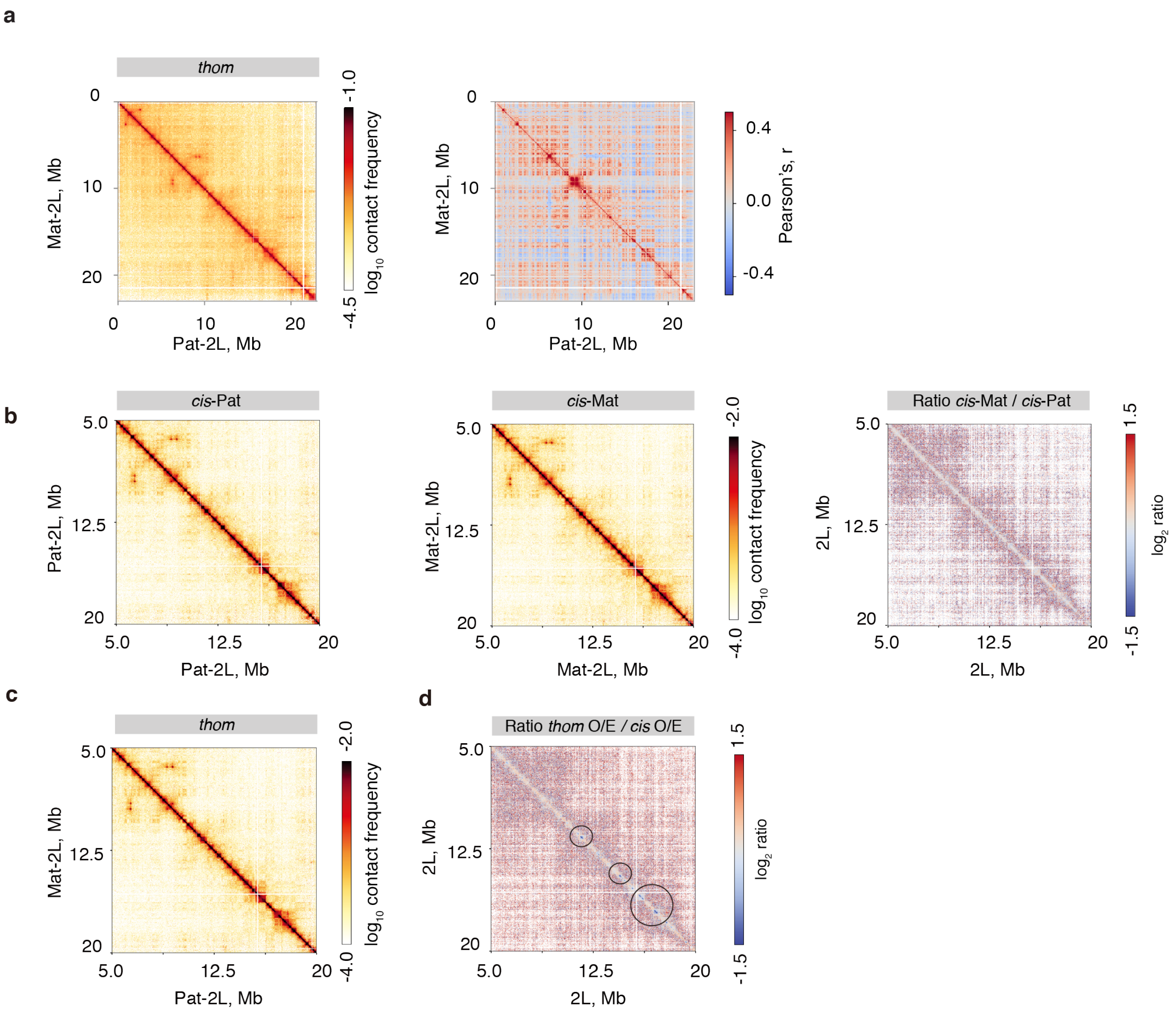

Supplementary Figure 4. Haplotype-resolved contact maps reveal compartments between homologs and concordance of *cis* and *thom* interactions. a, The *thom* contact map at 2L at 40 kb resolution and (left) a map of Pearson's correlation coefficients between rows and columns of the *thom* contact map (right). Both maps show a plaid-like pattern indicative of A-B compartmentalization of loci on homologous chromosomes. **b,** Paternal and maternal *cis* contact maps within 5-20 Mb at 2L are highly concordant. **c,** *Thom* contact map within 5-20 Mb at 2L. **d,** Regions as in b, and c showing *thom* contact frequency depletion detected in blue (circles pointing to some *thom* depleted regions).

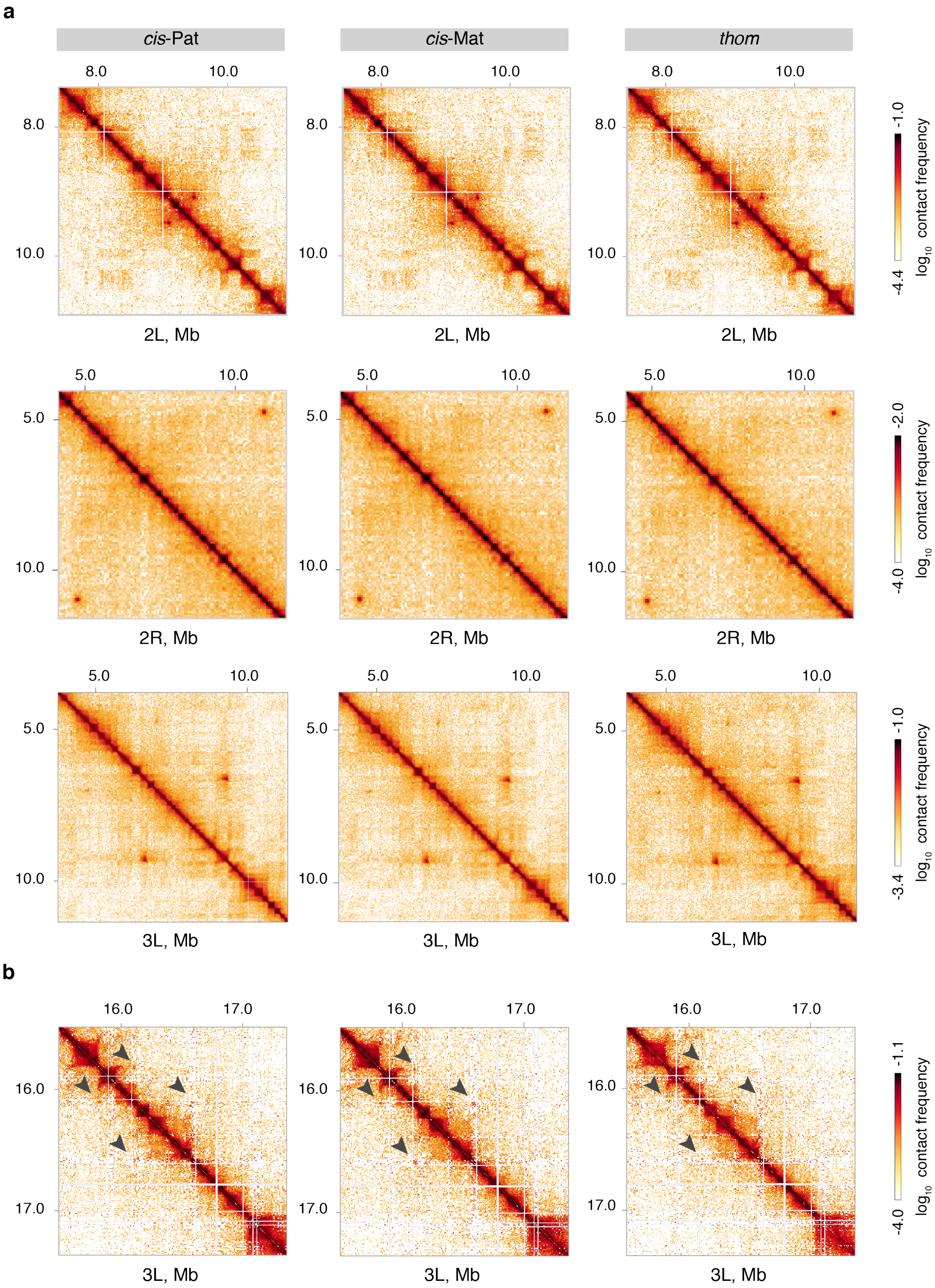

Supplementary Figure 5. **Examples of loops or long-range interaction peaks detected in *cis*-paternal and maternal contact maps, as well as *thom* contact maps a,** Examples in 2L, 2R, and 3L chromosome arms **b,** Examples of loops not detected in all three maps.

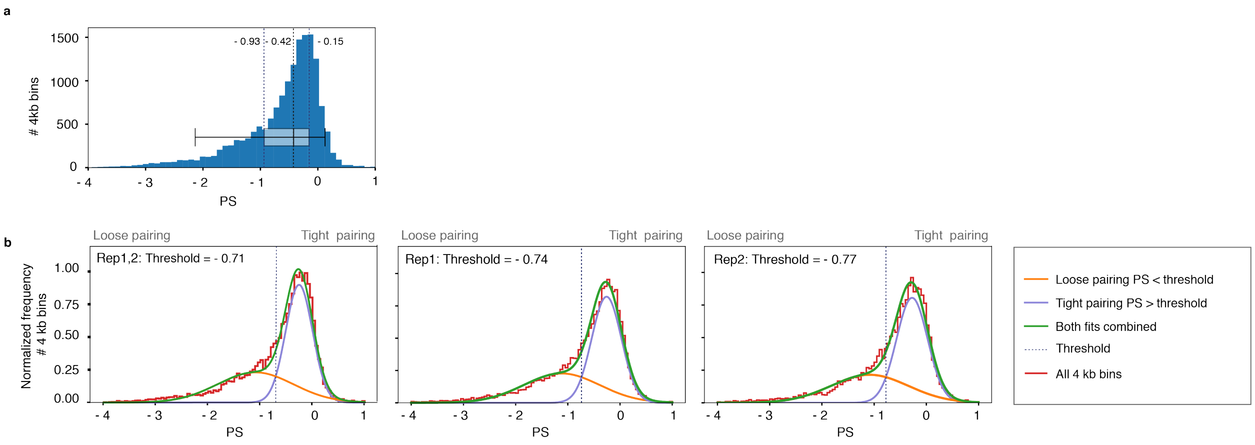

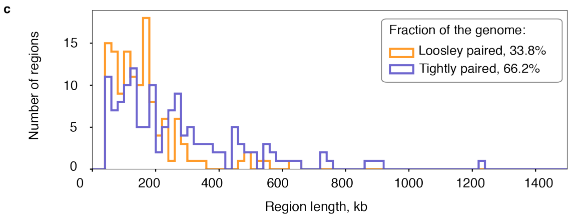

d

Sample	Tight	Loose
**Untreated cells (rep 1, 2)**	66.2	33.8
**Rep1**	66.6	33.4
**Rep2**	68.8	31.2

Supplementary Figure 6. **Pairing score distribution and breakdown into tight and loose regions in PnM genome. a,** Genome-wide distribution of pairing score (PS) in PnM shows a well-pronounced peak at higher values of PS and a tail extending into the lower values of PS. **b,** We interpret the PS distribution with a model, where each bin can be either tightly or loosely paired. We can separate the peak from the tail of the distribution by fitting it with a sum of two Gaussians. Left panel: All genomic bins with PS < -0.71 are considered loosely paired (since they are more likely to belong to the low-PS Gaussian) and the bins with PS > -0.71 are considered tightly paired. Two right panels: the Gaussian fitting procedure and the resulting thresholds were reproducible per replicate. **c,** The length distribution of loosely and tightly paired regions. **d,** The breakdown of tight and loose pairing based on the threshold produced comparable tight-to-loose ratios for each biological replicate.

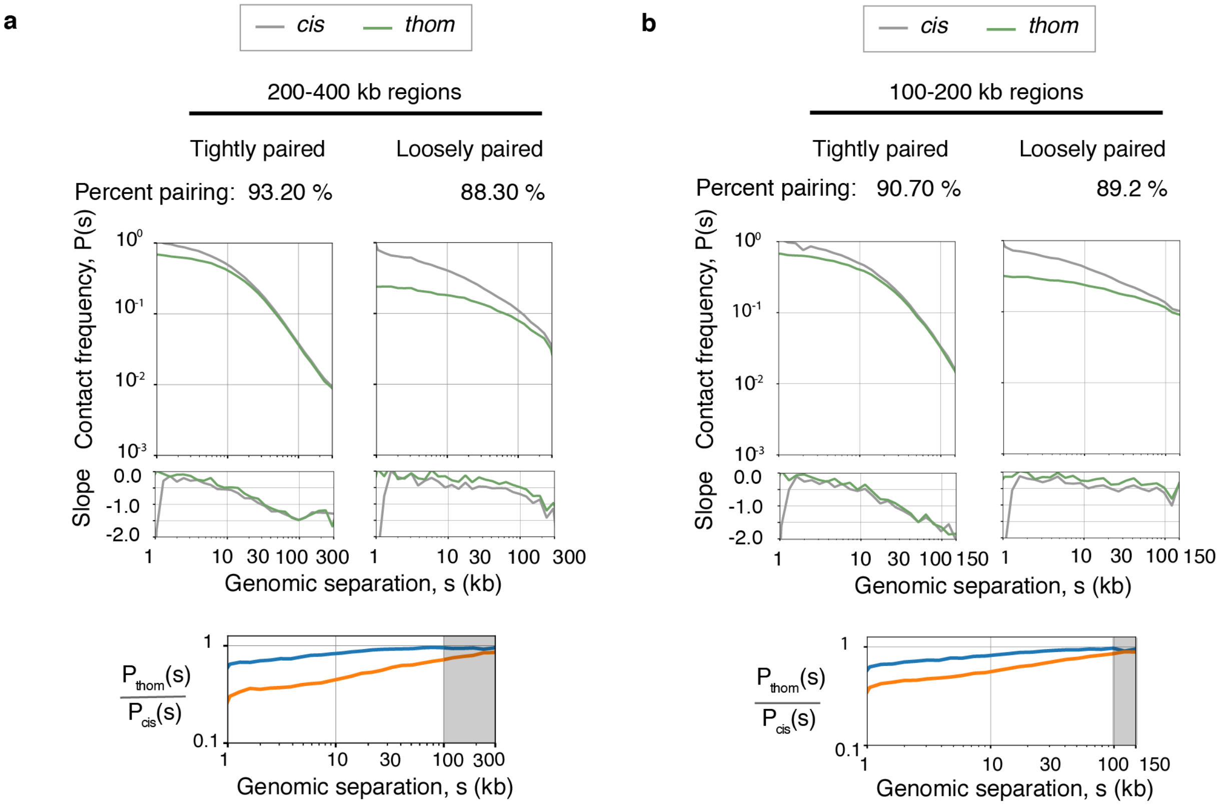

Supplementary Figure 7. $P_{thom}(s)$, $P_{cis}(s)$ for tight and loose regions of different sizes. Top, $P_{thom}(s)$, $P_{cis}(s)$, middle, slope, and bottom, is $P_{thom}(s)$/ $P_{cis}(s)$ contact frequency within tightly and loosely paired regions. **a,** 200-400 kb length and **b,** 100-200 kb length. In tighly paired regions, *thom* and *cis* contact frequency curves show two modes of decay, shallow and steep, while in loosely paired regions, the curves have only one, shallow mode. Shaded area shows the region used to determine the percent pairing in **a**, by calculating the geometric mean of the $P_{thom}(s)$/ $P_{cis}(s)$ at larger genomic separations (100-300 kb, or 100-150 kb).

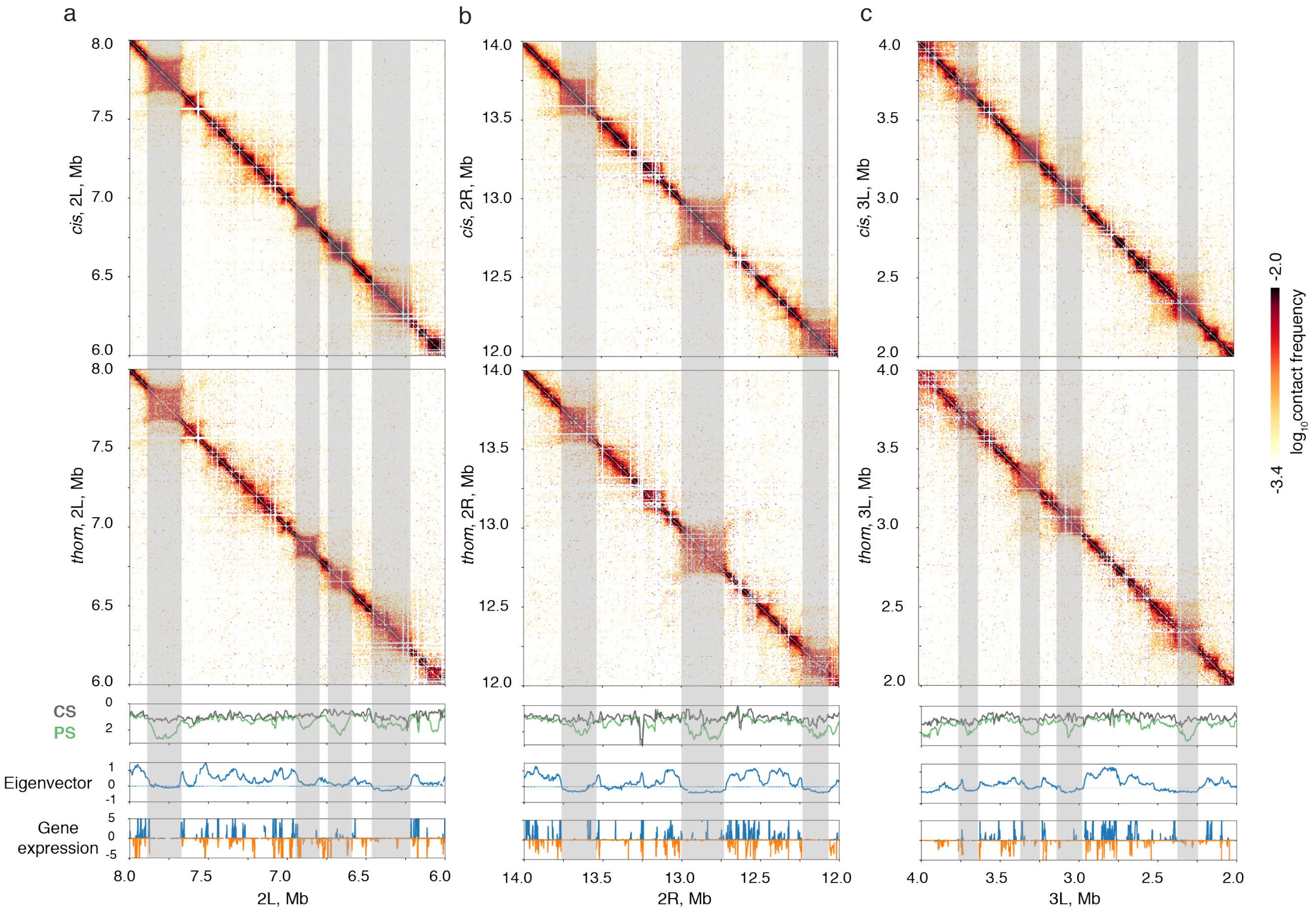

Supplementary Figure 8. Representative examples of pairing relative to genomic compartment type and gene expression. **a-c,** 2 Mb-long example regions described with *cis* and *trans*-homolog (*thom*) contact maps (two upper panels), pairing (PS) and *cis* scores (CS) (middle panel) and measures of transcriptional activity, eigenvector and RNA-seq (two lower panels). High PS correlates with high levels of gene expression, and enrichment of A-type compartments. Shaded area, shows some weakly paired regions, indicating a lower eigenvector rank, and generally lower expression levels. **a,** 2L, 6-8 Mb, **b,** 2R, 12-14 Mb and **c,** 3L, 2-4 Mb.

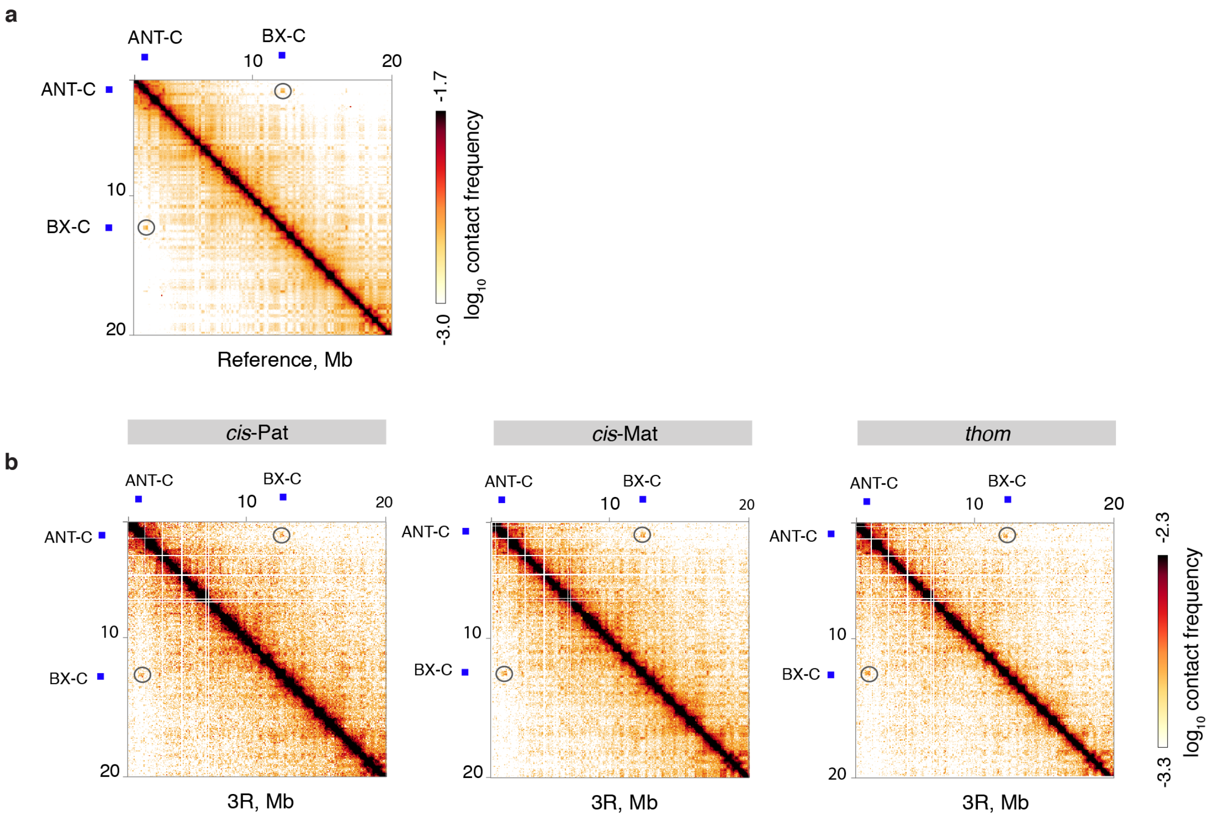

Supplementary Figure 9. *Thom* interaction of ANT-C and BX-C. a, Long-range *cis*-interactions that were detected in previous studies between ANT-C and BX-C[2,3] are detected in PnM reference map (non-allele-specific mapping), **b,** and *thom* and *cis* maps, indicating that this well-known interaction in *cis,* is also detected in *thom* maps, and is within the same compartment in our haplotype-maps.

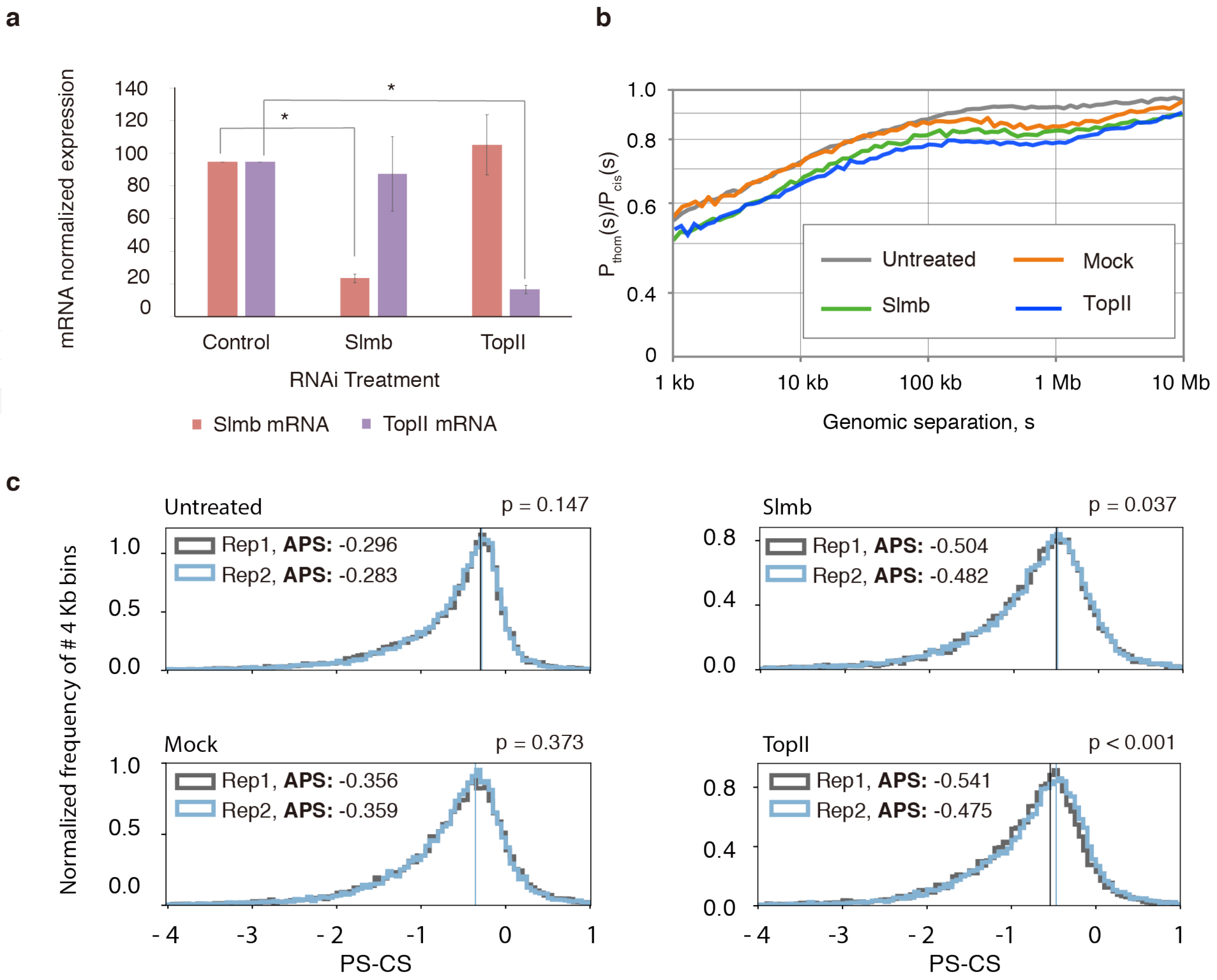

Supplementary Figure 10. The effect of knocking down Slmb or TopII. a, Quantitative PCR confirmed efficient knockdown of Slmb and TopII. The expression levels are normalized to Act5c and RP49 expression after RNAi in PnM cells. There is a significant drop in the levels of Slmb and TopII mRNA compared to the control mock treatment. Asterisks denote a significant reduction from control ($P < 0.0001$, unpaired t-test). S.d are shown for at least 3 biological replicates. **b,** The ratio of *thom*-to-*cis* contact frequency as a function of genomic separation, s, in Slmb, and TopII RNAi sample show a drop in *thom*-to-*cis* frequency compared to mock at distances below 100 kb and at all genomic separations compared to the untreated PnM cells. **c,** Aggregated pairing scores (APS) are reduced in Slmb and TopII RNAi samples compared to

untreated cells, and mock replicates, for two biological replicates as illustrated by a drop in the PS-CS distribution. P-values determined using bootstrapping (Supplementary methods).

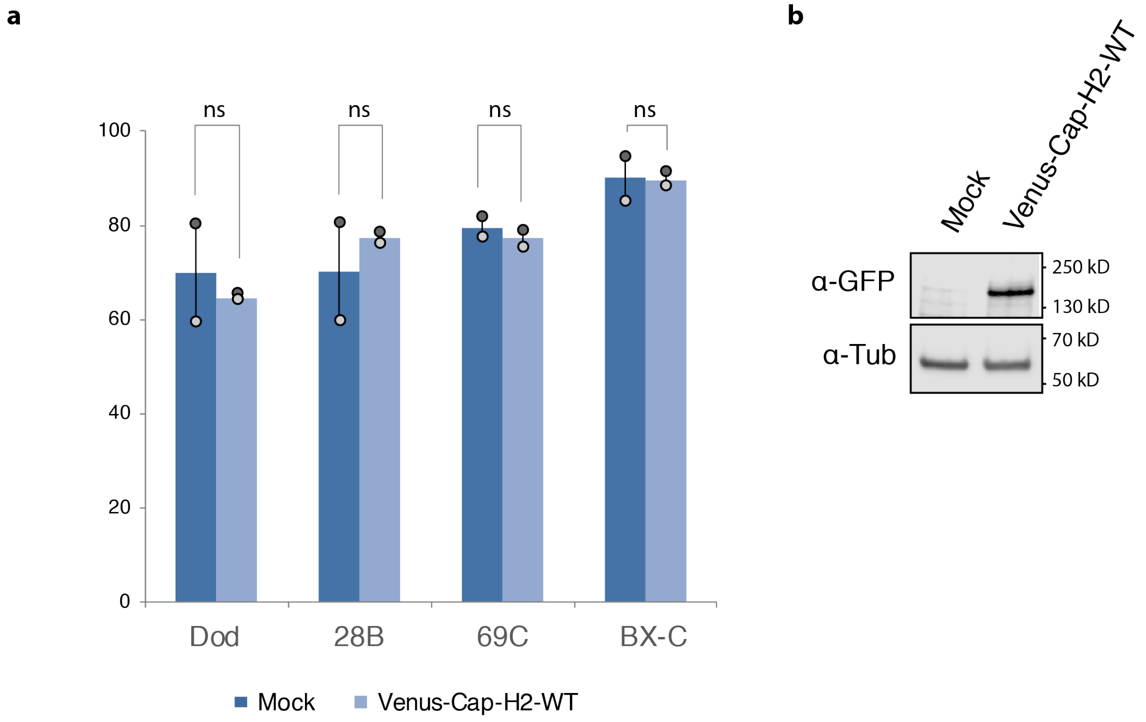

Supplementary Figure 11. Overexpression of Cap-H2 in PnM a, The effect of Cap-H2 overexpression on pairing as determined by FISH. Levels of pairing (quantified and displayed as in Figure 1d, in PnM cells after transient expression of venus-Cap-H2-WT). (P =ns, non-significant, unpaired t-test, error bars, s.d for two biological replicates; for N > 100 nuclei/replicate). **b,** Protein levels detected in the two samples, Mock, and venus-Cap-H2-WT. Source data are provided as a Source Data file.

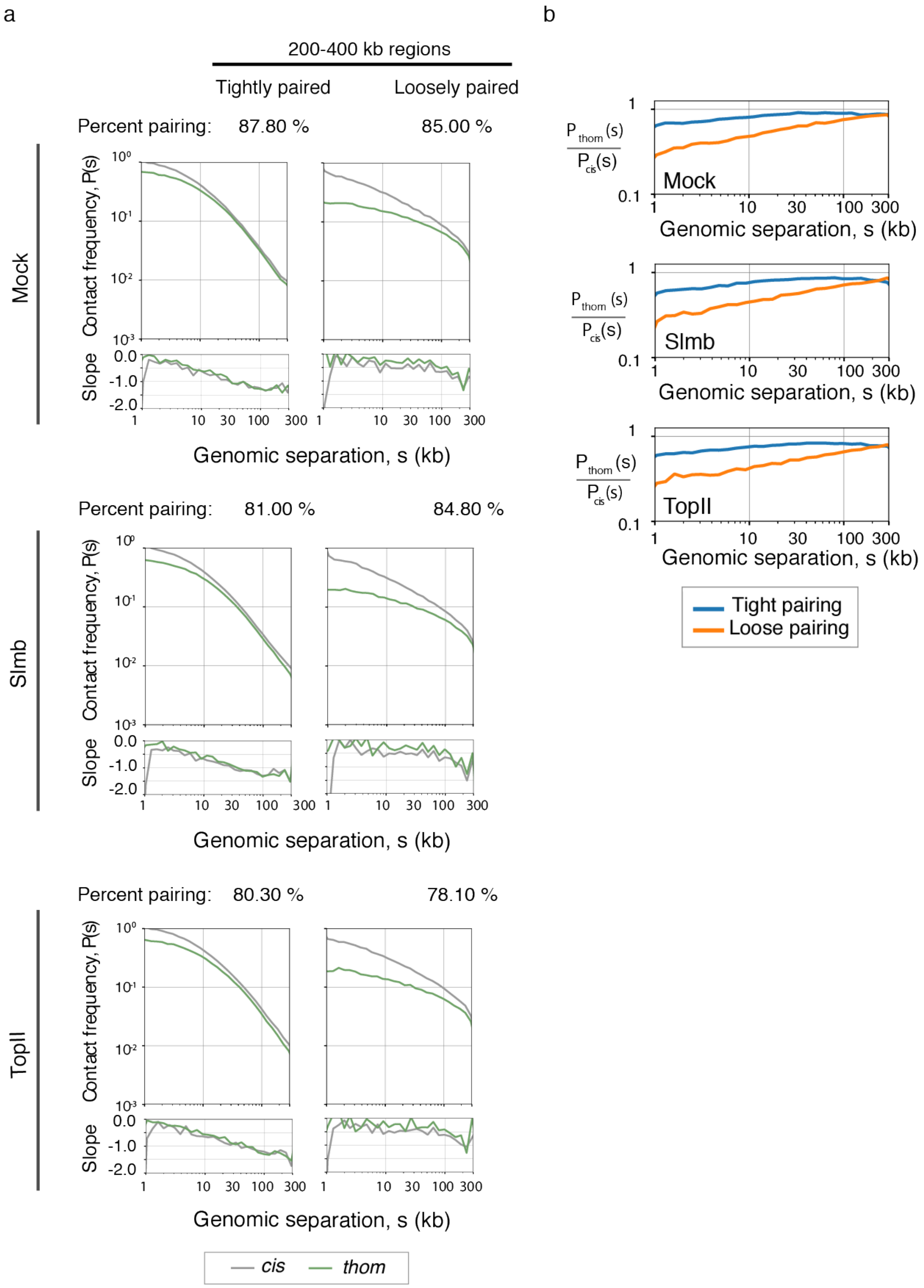

Supplementary Figure 12. The effect of knocking down Slmb or TopII on 200-400 kb regions of tight and loose pairing. a, Top, plots of $P(s)_{thom}$ and $P(s)_{cis}$, bottom, slopes, within tightly (left) and loosely paired (right) regions of 200-400 kb length. **b,** $P(s)_{thom}$ / $P(s)_{cis}$ relative

to genomic separation, s, in tightly and loosely paired regions. Shaded area shows the region used to determine the percent pairing in **a,** by calculating the geometric mean of the $P(s)_{thom}$ / $P(s)_{cis}$ at larger genomic separations of 100-300 kb. A comparison between tight and loose pairing *thom* and *cis* contact frequency shows a drop in both tight and loose pairing for Slmb and TopII relative to untreated cells.

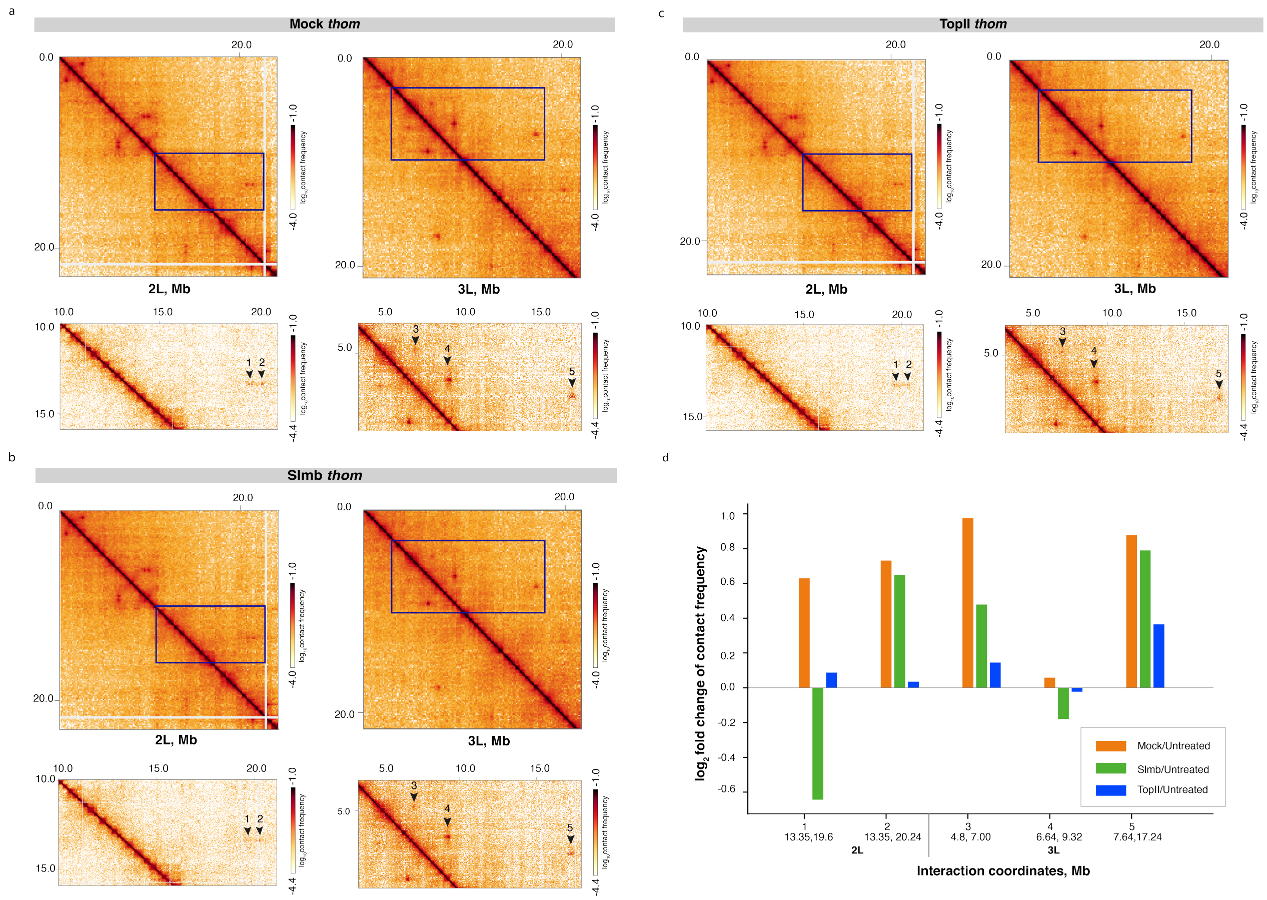

Supplementary Figure 13. *Thom* contact maps for Mock, Slmb or TopII conditions a-c, 2L and 3L whole chromosome arm *thom* contact maps (top) for **a,** Mock, **b,** Slmb RNAi, **c,** TopII RNAi, and zoom-ins (bottom). Arrows point to long range contacts numbered 1-5, some of which are reduced in the knockdowns as compared to mock. **d,** Quantification of the interactions labeled 1-5 in the *thom* maps (a-c) relative to untreated samples. The contact frequency at each interaction peak was calculated as the sum of the Iteratively Corrected Contact Frequency in a 120 kb x120 kb window surrounding it.

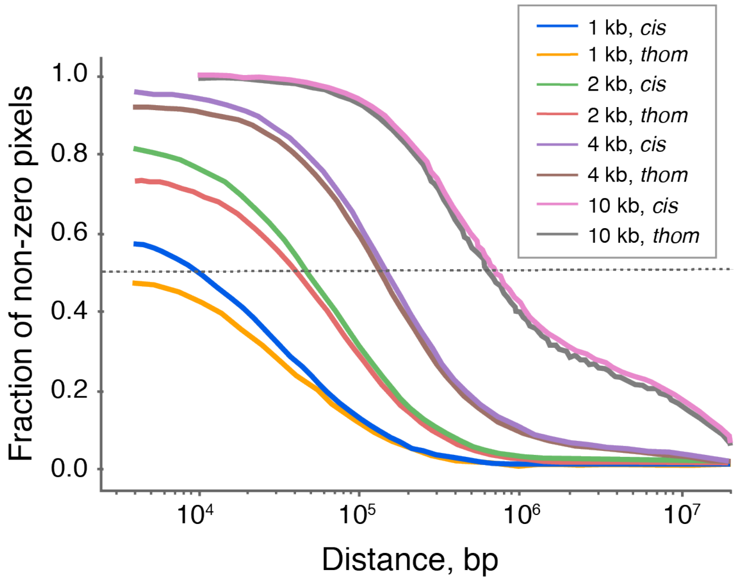

Supplementary Figure 14. Determining optimal resolution for *cis* and *thom* contact maps. The curves show the fraction of non-zero pixels (y-axis) at various genomic separations (x-axis) in *cis* and *thom* contact maps of various resolutions. We find that the 4 kb resolution maps have <50% empty pixels (the horizontal dashed line) at genomic separations corresponding to the TAD interactions (<~100kb).

Supplementary Tables

		Indels			SNVs			
**Chrom**	**Chrom size**	**#057**	**#439**	**hom**	**#057**	**#439**	**hom**	**het SNVs/kb**
**chr2L**	23011544	5820	5910	9415	66894	70989	68091	5.99
**chr2R**	21146708	5144	5396	8828	55659	58689	60606	5.41
**chr3L**	24543557	6068	5599	10374	67299	64261	73011	5.36
**chr3R**	27905053	6494	6402	9676	66777	68709	65627	4.86
**chr4**	1351857	1	4	136	2	15	929	0.01
**chrX**	22422827	8405	8386	5193	40674	44055	38063	3.78

Supplementary Table 1. Summary of SNV frequency per chromosome

	Untreated	RNAi Treatment		
	PnM	Mock	Slmb	TopII
**Total reads (x1e6)**	604.12	167.46	158.14	164.81
**Total non-haplotype-specific mapped reads (x1e6)**	411.00	116.87	108.68	115.22
**% Non-haplotype-specific mappability**	68.03	69.79	68.72	69.91
**Total haplotype-specific mapped reads (x1e6)**	75.38	23.12	21.51	22.98
**% Haplotype-specific mappability**	12.48	13.80	13.60	13.94
***Cis* reads (x1e6)**	55.44	16.86	15.80	17.09
***Trans* reads (x1e6)**	19.95	6.25	5.72	5.89
**Ratio *cis:trans***	2.78	2.70	2.76	2.90
**% *Cis* of total sequenced reads**	9.18	10.07	9.54	10.37
**% *Trans* of total sequenced reads**	3.30	3.73	3.62	3.57
**Total haplotype-specific mapped reads without X (x1e6)**	45.95	14.08	13.16	13.89

Supplementary Table 2. Summary of non-haplotyped (reference-mapped), and haplotype-specific read pairs recovered for untreated PnM and RNAi treated cells, with two biological replicates merged per sample.

ChIP-seq	Percent boundaries overlapping ChIP peaks	Correlation ChIP-seq fold change over input vs pairing score, r_s	Cell type	Accession number
**Nup98**	70.7	0.467	Kc167	GSM2133770
**RNAPII**	71.1	0.462	Kc167	GSM1536014
**GAF**	25.8	0.459	Kc167	GSM2133762
**GAF**	54.7	0.310	Kc167	GSM1318358
**GAF**	41.4	0.116	S2	GSM998826
**BEAF**	58.4	0.452	Kc167	GSM1535963
**BEAF**	52.8	0.277	Kc167	GSM807545
**BEAF**	50.4	0.250	Kc167	GSM762845
**Cap-H2**	59.6	0.449	Kc167	GSM1535966, 67
**TFIIIC**	50.1	0.448	Kc167	GSM1536019, 20
**Mrg15**	48.1	0.439	S2	GSM2443790,91
**Fs1h**	32.5	0.422	Kc167	GSM1535987, 88
**Mod**	32.9	0.388	Kc167	GSM892321, 22
**CP190**	77.6	0.384	Kc167	GSM1535980
**CP190**	77.1	0.342	Kc167	GSM807541
**CP190**	76.9	0.184	Kc167	GSM762836
**CBP**	78.4	0.372	Kc167	GSM1535970, 71
**ZIPIC**	66.3	0.368	Kc167	GSM2133769
**Cohesin**	76.1	0.348	Kc167	GSM1536009, 10, 11
**Ibf2**	45.1	0.308	Kc167	GSM2133766
**Ibf1**	54.8	0.287	Kc167	GSM2133767
**Pita**	40.0	0.281	Kc167	GSM2133768
**Chromator**	45.3	0.280	Kc167	GSM1318357
**Chromator**	53.5	0.250	Kc167	GSM1535975, 76
**CTCF**	23.9	0.208	Kc167	GSM807543
**CTCF**	51.9	0.174	Kc167	GSM762842
**CTCF**	13.6	-0.068	Kc167	GSM1535983
**DREF**	36.5	0.200	Kc167	GSM1535984, 85
**DREF**	46.8	-0.149	Kc167	GSM977023, 24
**Su(Hw)**	50.3	0.057	Kc167	GSM762839

Supplementary Table 3. The summary of the overlap between PnM boundaries and ChIP-seq peaks from published data sets, and their correlation with tight pairing**.** ChIP-seqs are arranged based on their correlation coefficient (r_s , Spearman's), red to blue color assigned from highest

(0.467) to lowest (-0.149) r_s values . All reported correlations have p-values $P < 0.0001$. Discrepancies in the correlations for the same protein across different data sets could be due antibody quality, type of cells used, or different cell batches used in different labs.

Locus	Quadrant	Expression in PnM	Pairing	Assays confirming transvection
dpp	1	yes	tight	*trans*-allelic complementation[4]
eya	1,2	yes/low	tight	*trans*-allelic complementation[5]
Gpdh1	2	no	tight	*trans*-allelic complementation[6]
esg	3,4	yes/low	loose	transgene insertion at endogenous locus and PSS[7]
ap	1	yes	tight	*trans*-allelic complementation[8]
eve	2	no	tight	*trans*-homing, transgene insertion, PSS, Lac*Z* expression[9], H3C[10]
inv	3,4	yes/low	loose	transgene insertion, PSS, Lac*Z* expression in discs[11]
en	1	yes	tight	transgene insertion, PSS, Lac*Z* expression in embryos, imaginal discs, p-element homing [7,12,13]
vg	1	yes	tight	*trans*-allelic complementation[14], transgene insertion[15] .
mbl	1,2	yes/low	tight	*trans*-allelic complementation[16]
Men	1,2	no	tight	*trans* -allelic complementation[17,18]
bw	1,2	yes/low	tight	transgene insertion, PEV, PSS[19-22]
Scr	1,2	no	tight	*trans* -allelic complementation[23,24]
ss	2	no	tight	transgene insertion and expression in photoreceptors[25]
Ubx	2,3	very low	tight, loose	*trans*-allelic complementation[26-29]
abd-A	2,3	very low	tight, loose	*trans*-allelic complementation, transgene insertion, PSS, Lac*Z* expression[30]
Abd-B	2	very low	tight	*trans* -allelic complementation[30-33]

Supplementary Table 4. Examples of known transvected loci on the autosomes and their location in Fig. 3d. Quadrant 1, and 2, represents regions in the genome that are tightly paired and are expressed or not expressed at all, respectively. *Pairing sensitive silencing (PSS), high resolution chromosome conformation capture (H3C), position effect variegation (PEV). *The summary of loci above includes at least one endogenous locus study, or endogenous locus regulatory region.*

Assay	Slmb	TopII
qPCR showing mRNA depletion vs. mock	75.2 %	82.5 %
Average FISH drop in colocalization vs. mock	18.47 %	14.07 %
Average FISH drop in colocalization vs. untreated sample	30.16 %	27.35 %
APS change vs. mock	9.46 %	8.95 %
APS change vs. untreated sample	13.29 %	12.80 %

Supplementary Table 5. Summary of different pairing assays for Slmb and TopII knockdown samples relative to mock sample. APS: aggregated pairing score.

Probe target	dm3 Coordinates	OligoLibraryID	Total # probes	Target size (Mb)	Secondary oligo ID used
**28B**	chr2L: 7,256,488-7,936,487	Mycroarray-6151	10000	0.680	Sec5
**69C**	chr3L: 12,170,682-12,844,681	Mycroarray-6150	10000	0.674	Sec1
**BX-C**	chr3R: 12,482,502-12,797,965	Mycroarray-6010	2394	0.316	Sec6
**16E**	chrX: 17,406,557-18,106,556	Mycroarray-6148	10000	0.700	Sec6
**HOPs 2L**	chr2L:10,731-1,999,862	Mycroarray	2410	2.000	2L-057: Sec6, 2L-439: Sec5
**HOPs 3L**	chr3L: 19,809-1,999,387	Mycroarray	2304	2.000	3L-057: Sec6, 3L-439: Sec5

Supplementary Table 6. Oligopaints probe target library summary

Name	Primer (5’-3’)
**28B-F**	TAGCGCAGGAGGTCCACGACGTGCAAGGGTGTCGCTCGGTCTCCGTTCGTCTC
**28B-R**	TAATACGACTCACTATAGGGGGGCTAGGTACAGGGTTCAGC
**69C-F**	CACCGACGTCGCATAGAACGGAAGAGCGTGTGCGCTCGGTCTCCGTTCGTCTC
**69C-R**	TAATACGACTCACTATAGGGGGGCTAGGTACAGGGTTCAGC
**BX-C-F**	CACACGCTCTCCGTCTTGGCCGTGGTCGATCAGTATCGTGCAAGGGTGAATGC
**BX-C-R**	TAATACGACTCACTATAGGGGAGCAGTCACAGTCCAGAAGG
**16E-F**	CACACGCTCTCCGTCTTGGCCGTGGTCGATCACGCTCGGTCTCCGTTCGTCTC
**16E-R**	TAATACGACTCACTATAGGGGGGCTAGGTACAGGGTTCAGC
**HOPs-057c-2L-F**	CACACGCTCTCCGTCTTGGCCGTGGTCGATCAATGCGTTCGGTCTCCGTCAAC
**HOPs-057c-2L-R**	TAATACGACTCACTATAGGGAATCGCGACGTGTGATGGAAC
**HOPs-057w-2L-F**	CACACGCTCTCCGTCTTGGCCGTGGTCGATCAATTTGTCGACCCGAGTCGAAC
**HOPs-057w-2L-R**	TAATACGACTCACTATAGGGAACCGGTCGGGATTCCGTAAC
**HOPs-439c-2L-F**	TAGCGCAGGAGGTCCACGACGTGCAAGGGTGTAACATACGCCTCGGGTTGGAC
**HOPs-439c-2L-R**	TAATACGACTCACTATAGGGAGTGTCGCGTCGGCCAGAAAC
**HOPs-439w-2L-F**	TAGCGCAGGAGGTCCACGACGTGCAAGGGTGTACCCGATACGTCGTGGGATTC
**HOPs-439w-2L-R**	TAATACGACTCACTATAGGGACTCGGCGTCTTCCGACGATG
**HOPs-057c-3L-F**	CACACGCTCTCCGTCTTGGCCGTGGTCGATCAACCGGGTCCCGTACATTTGCC
**HOPs-057c-3L-R**	TAATACGACTCACTATAGGGATGGTAACGCAACGGATCTCG
**HOPs-057w-3L-F**	CACACGCTCTCCGTCTTGGCCGTGGTCGATCAACATTCGCGCACGCTAATGTC
**HOPs-057w-3L-R**	TAATACGACTCACTATAGGGAAGCGGCGTTCGACACCTTTG
**HOPs-439c-3L-F**	TAGCGCAGGAGGTCCACGACGTGCAAGGGTGTAGAGGGCGGTGCGGTACTAAG
**HOPs-439c-3L-R**	TAATACGACTCACTATAGGGACCGTCAGGTCGACGCTACAC
**HOPs-439w-3L-F**	TAGCGCAGGAGGTCCACGACGTGCAAGGGTGTAAGGAGCGTCCGCACCGAATG
**HOPs-439w-3L-R**	TAATACGACTCACTATAGGGACATTGGGTGCGATGACGAAC

Supplementary Table 7. PCR primers for amplifying Oligopaints libraries

Target	flyRNAi ID#		Primer (5’-3’)
**Slmb**	DRSC32609	Fwd	TAATACGACTCACTATAGGGAGAAAGCAGGAGCCGGTGAA
		Rev	TAATACGACTCACTATAGGGAGACAAAAGGCATCTGGTCTTCT
**TopII**	DRSC03459	Fwd	TAATACGACTCACTATAGGGTTTGCCAGAGCGATATCTC
		Rev	TAATACGACTCACTATAGGGCCATAGTGGCTCGATCTTTT

Supplementary Table 8. dsRNA synthesis primers

Name	Primer (5’-3’)
**Act5C-F**	CACCGGTATCGTTCTGGACT
**Act5C-R**	AGGGCAACATAGCACAGCTT
**RP49-F**	CCGCTTCAAGGGACAGTATC
**RP49-R**	GACAATCTCCTTGCGCTTCT
**Slmb-F**	ACACCCTTATCCACCACTGC
**Slmb-R**	CAAAGTCCACCACATTGACG
**TopII-F**	CGCAAGTCAAGCAAGATCAA
**TopII-R**	GCTTCCCGCACATTTAGAAG

Supplementary Table 9. qPCR primers

	PnM genome	Maternal genome (DGRP-057)	Paternal genome (DGRP-439)
Number of raw read pairs	154,862,547	63,470,151	62,515,671
Number of mapped, paired, and de-duplicated read pairs	131,879,436	58,974,713	58,287,559

Supplementary Table 10. Summary of the number of read pairs recovered from sequencing the PnM hybrid, maternal, and paternal genome libraries and used for phasing the hybrid-haplotype PnM genome and determining SNVs *de novo*.

Type of reads	Rep1	Rep2
cis	27,500,435	28,017,074
cis 3kb	16,314,399	16,908,792
cis 10kb	14,978,208	15,590,307
cis 100kb	10,247,013	10,738,792
cis 2L, 2R, 3L	10,467,549	10,608,473
cis 2L, 2R, 3L 3kb	4,927,548	5,105,874
cis 2L, 2R, 3L 10kb	4,405,081	4,590,017
cis 2L, 2R, 3L 100kb	2,692,950	2,837,605
thom	5,822,100	6,034,950
thom 3kb	5,548,717	5,769,358
thom 10kb	5,097,220	5,322,011
thom 100kb	3,299,001	3,478,983
thom 2L, 2R, 3L	4,466,718	4,629,258
thom 2L, 2R, 3L 3kb	4,254,561	4,423,056
thom 2L, 2R, 3L 10kb	3,901,426	4,072,986
thom 2L, 2R, 3L 100kb	2,516,559	2,652,904
thet	3,961,794	4,413,743

Supplementary Table 11. Breakdown of haplotype-resolved read pairs at different genomic separation per replicate. *thom*: *trans* homologous, *thet*: *trans* heterologous

Supplementary Note 1

Dividing the genome into regions of tight and loose pairing, and $P(s)^{tight}$ and $P(s)^{loose}$ curves

For each type of pairing, we calculated P(s) curves over large regions with a relatively narrow size range of 200-400 kb, or 100-200 kb (Supplementary Fig. 7). Within 200-400 kb tightly paired regions, the $P_{cis}(s)$ and $P_{thom}(s)$ curves showed two modes: (i) a shallow mode at shorter separations < 30kb, where $P_{thom}(s)$ was noticeably lower than $P_{cis}(s)$ (at 1-3 kb, $P_{thom}(s)/P_{cis}(s)$ is 0.66), and where both decayed relatively slowly with distance ($P(s) \sim s^{\alpha}$, α ~0.25-0.5); and (ii) a steep mode at larger separations > 30 kb, where the two curves were almost equal ($P_{thom}(s)/P_{cis}(s)$= 0.93) and decayed more rapidly ($P(s) \sim s^{\alpha}$, α~1.0-1.5) (Fig. 2h, left panel). In loosely paired regions, $P_{thom}(s)$ and $P_{cis}(s)$ had only one shallow mode, where $P_{thom}(s) < P_{cis}(s)$ at all separations, (Fig. 2h, right panel); and both curves decayed slowly ($P(s) \sim s^{\alpha}$, α ~0.25-0.5). We observed similar behavior of $P_{cis}(s)$ and $P_{thom}(s)$ in regions of 100-200 kb (Supplementary Fig. 7b).

In *cis*, the existence of the shallow mode in the $P_{cis}(s)$ curves reflects formation of domains, as the slow decay of contact frequency with distance (100X increase in distance leads to only 10X drop in contact frequency) is inconsistent with random folding of the chromatin[34,35]. Importantly, the transition into the steep mode occurs at the average domain size[34]. Thus, our results suggested that an individual tightly paired region in *cis* consists of a series of smaller domains (~10-30 Kb), while an individual loosely paired region could correspond to a single domain.

We used the deviation of $P_{thom}(s)$ from $P_{cis}(s)$ in tightly and loosely paired regions to infer the precision of homologous pairing. If two homologous chromosomes were connected with each other at every base pair (either by direct base-pair-complementarity or by a sequence-specific binding protein or other mechanism), $P_{thom}(s)$ would equal $P_{cis}(s)$ at all separations. However, if homologous loci were linked with each other intermittently, pairs of loci at shorter distances would contact less frequently in *thom* than in *cis* ($P_{thom}(s) < P_{cis}(s)$), and at sufficiently large separations pairs of loci would contact each other as often in *thom* as in *cis* ($P_{thom}(s) = P_{cis}(s)$). In our data, in tight regions, *thom* and *cis* contacts approached each other in frequency at s = ~10-30 kb, and, in loose regions, only for loci located on the ends of the region (i.e. at s ~150 kb for 100-200 kb regions and at s ~300 kb for 200-400 kb regions) (Supplementary Fig. 7). Thus, we concluded that our data was consistent with a model, where tightly paired regions are connected in *thom* every ~10-30 kb, within the average domain size in these regions, and probably at domain boundaries, and loosely paired regions are connected only at their boundaries. This allowed us to hypothesize that (a) the difference between tightly and loosely paired region is due to higher frequency of *thom* connections, in tightly paired regions (b) pairing at loose regions is affected by pairing at the flanking tight regions. To conclude, within our resolution limit (~16 kb), and given that in tightly paired regions, *thom* contacts at the highest registration appeared as frequent as *cis* contacts at s = ~5 kb and, in loose regions, the frequency of such *thom* contacts matched that of *cis* contacts at s = ~30 kb (Fig. 2h), pairing in tight regions is more precise, likely due to more frequent connections between homologs within tight regions, possibly at domain boundaries. Furthermore, we found that *thom* contact frequency approached 93.2% of *cis* in tightly paired regions (at s = 100-300 kb) (defined as a geometric mean of

thom/cis contact frequency at s =100-300kb), suggesting that as many as 93.2% of cells in our sample are exhibiting pairing at those regions (Supplementary Fig. 7). The fraction of cells without pairing, could represent a population of cells that are undergoing mitosis. Using the same approach, we found that the fraction of entirely unpaired tight regions in Slmb and TopII knockdowns increased by 6.80% and 7.50%, relative to mock, and 12.2% and 12.9%, relative to untreated sample (Supplementary Fig. 12).

Supplementary References

1 Erceg, J. *et al.* The genome-wide, multi-layered architecture of chromosome pairing in early Drosophila embryos *bioRxiv. Preprint at https://www.biorxiv.org/content/10.1101/443028v1* (2018).

2 Sexton, T. *et al.* Three-dimensional folding and functional organization principles of the Drosophila genome. *Cell* **148**, 458-472, doi:10.1016/j.cell.2012.01.010 (2012).

3 Bantignies, F. *et al.* Polycomb-dependent regulatory contacts between distant Hox loci in Drosophila. *Cell* **144**, 214-226, doi:10.1016/j.cell.2010.12.026 (2011).

4 Gelbart, W. M. Synapsis-dependent allelic complementation at the decapentaplegic gene complex in Drosophila melanogaster. *Proc Natl Acad Sci U S A* **79**, 2636-2640 (1982).

5 Leiserson, W. M., Bonini, N. M. & Benzer, S. Transvection at the eyes absent gene of Drosophila. *Genetics* **138**, 1171-1179 (1994).

6 Gibson, J. B. R., D. S.; Bartoszewski, S,; Wilks, A. V. Structural Changes in the Promoter Region Mediate Transvection at the sn-Glycerol-3-Phosphate Dehydrogenase Gene of Drosophila melanogaster. *Biochemical Genetics* **37**, 301-315 (1999).

7 Kassis, J. A. Unusual properties of regulatory DNA from the Drosophila engrailed gene: three "pairing-sensitive" sites within a 1.6-kb region. *Genetics* **136**, 1025-1038 (1994).

8 Gohl, D., Muller, M., Pirrotta, V., Affolter, M. & Schedl, P. Enhancer blocking and transvection at the Drosophila apterous locus. *Genetics* **178**, 127-143, doi:10.1534/genetics.107.077768 (2008).

9 Fujioka, M., Wu, X. & Jaynes, J. B. A chromatin insulator mediates transgene homing and very long-range enhancer-promoter communication. *Development* **136**, 3077-3087, doi:10.1242/dev.036467 (2009).

10 Fujioka, M., Mistry, H., Schedl, P. & Jaynes, J. B. Determinants of Chromosome Architecture: Insulator Pairing in cis and in trans. *PLoS Genet* **12**, e1005889, doi:10.1371/journal.pgen.1005889 (2016).

11 Cunningham, M. D., Brown, J. L. & Kassis, J. A. Characterization of the polycomb group response elements of the Drosophila melanogaster invected Locus. *Mol Cell Biol* **30**, 820-828, doi:10.1128/mcb.01287-09 (2010).

12 Kassis, J. A., VanSickle, E. P. & Sensabaugh, S. M. A fragment of engrailed regulatory DNA can mediate transvection of the white gene in Drosophila. *Genetics* **128**, 751-761 (1991).

13 Cheng, Y., Kwon, D. Y., Arai, A. L., Mucci, D. & Kassis, J. A. P-element homing is facilitated by engrailed polycomb-group response elements in Drosophila melanogaster. *PLoS One* **7**, e30437, doi:10.1371/journal.pone.0030437 (2012).

14 Coulthard, A. B., Nolan, N., Bell, J. B. & Hilliker, A. J. Transvection at the vestigial locus of Drosophila melanogaster. *Genetics* **170**, 1711-1721, doi:10.1534/genetics.105.041400 (2005).

15 Schoborg, T., Kuruganti, S., Rickels, R. & Labrador, M. The Drosophila gypsy insulator supports transvection in the presence of the vestigial enhancer. *PLoS One* **8**, e81331, doi:10.1371/journal.pone.0081331 (2013).

16 Juni, N. & Yamamoto, D. Genetic analysis of chaste, a new mutation of Drosophila melanogaster characterized by extremely low female sexual receptivity. *J Neurogenet* **23**, 329-340, doi:10.1080/01677060802471601 (2009).
17 Merritt, T. J., Duvernell, D. & Eanes, W. F. Natural and synthetic alleles provide complementary insights into the nature of selection acting on the Men polymorphism of Drosophila melanogaster. *Genetics* **171**, 1707-1718, doi:10.1534/genetics.105.048249 (2005).
18 Bing, X., Rzezniczak, T. Z., Bateman, J. R. & Merritt, T. J. Transvection-based gene regulation in Drosophila is a complex and plastic trait. *G3 (Bethesda)* **4**, 2175-2187, doi:10.1534/g3.114.012484 (2014).
19 Sass, G. L. & Henikoff, S. Pairing-dependent mislocalization of a Drosophila brown gene reporter to a heterochromatic environment. *Genetics* **152**, 595-604 (1999).
20 Csink, A. K. & Henikoff, S. Genetic modification of heterochromatic association and nuclear organization in Drosophila. *Nature* **381**, 529-531, doi:10.1038/381529a0 (1996).
21 Csink, A. K., Bounoutas, A., Griffith, M. L., Sabl, J. F. & Sage, B. T. Differential gene silencing by trans-heterochromatin in Drosophila melanogaster. *Genetics* **160**, 257-269 (2002).
22 Henikoff, S. & Dreesen, T. D. Trans-inactivation of the Drosophila brown gene: evidence for transcriptional repression and somatic pairing dependence. *Proc Natl Acad Sci U S A* **86**, 6704-6708 (1989).
23 Pattatucci, A. M. & Kaufman, T. C. The homeotic gene Sex combs reduced of Drosophila melanogaster is differentially regulated in the embryonic and imaginal stages of development. *Genetics* **129**, 443-461 (1991).
24 Southworth, J. W. & Kennison, J. A. Transvection and silencing of the Scr homeotic gene of Drosophila melanogaster. *Genetics* **161**, 733-746 (2002).
25 Johnston, R. J., Jr. & Desplan, C. Interchromosomal communication coordinates intrinsically stochastic expression between alleles. *Science* **343**, 661-665, doi:10.1126/science.1243039 (2014).
26 Lewis, E. B. The Theory and Application of a New Method of Detecting Chromosomal Rearrangements in Drosophila melanogaster. *The American Naturalist* **88**, 225-239 (1954).
27 Gemkow, M. J., Verveer, P. J. & Arndt-Jovin, D. J. Homologous association of the Bithorax-Complex during embryogenesis: consequences for transvection in Drosophila melanogaster. *Development* **125**, 4541-4552 (1998).
28 Hartl, T. A., Smith, H. F. & Bosco, G. Chromosome alignment and transvection are antagonized by condensin II. *Science* **322**, 1384-1387, doi:10.1126/science.1164216 (2008).
29 Martinez-Laborda, A., Gonzalez-Reyes, A. & Morata, G. Trans regulation in the Ultrabithorax gene of Drosophila: alterations in the promoter enhance transvection. *Embo j* **11**, 3645-3652 (1992).
30 Lewis, E. B. A gene complex controlling segmentation in Drosophila. *Nature* **276**, 565-570 (1978).
31 Ronshaugen, M. & Levine, M. Visualization of trans-homolog enhancer-promoter interactions at the Abd-B Hox locus in the Drosophila embryo. *Dev Cell* **7**, 925-932, doi:10.1016/j.devcel.2004.11.001 (2004).
32 Hagstrom, K., Muller, M. & Schedl, P. A Polycomb and GAGA dependent silencer adjoins the Fab-7 boundary in the Drosophila bithorax complex. *Genetics* **146**, 1365-1380 (1997).
33 Muller, M., Hagstrom, K., Gyurkovics, H., Pirrotta, V. & Schedl, P. The mcp element from the Drosophila melanogaster bithorax complex mediates long-distance regulatory interactions. *Genetics* **153**, 1333-1356 (1999).
34 Fudenberg, G., Abdennur, N., Imakaev, M., Goloborodko, A. & Mirny, L. A. Emerging Evidence of Chromosome Folding by Loop Extrusion. *Cold Spring Harb Symp Quant Biol*, doi:10.1101/sqb.2017.82.034710 (2018).
35 Schwarzer, W. *et al.* Two independent modes of chromatin organization revealed by cohesin removal. *Nature* **551**, 51-56, doi:10.1038/nature24281 (2017).
