# Supplementary material for: Highly structured homolog pairing reflects functional organization of the Drosophila genome
Source: Nat Commun. 2019 Oct 3;10:4485. doi: 10.1038/s41467-019-12208-3 (PMC6776532; doi:10.1038/s41467-019-12208-3)
Supplement: Supplementary file 3 — Reporting Summary [file 41467_2019_12208_MOESM3_ESM.pdf]

nature research

Corresponding author(s): Leonid A. Mirny & Ting (C.-ting) Wu

Reporting Summary

Nature Research wishes to improve the reproducibility of the work that we publish. This form provides structure for consistency and transparency in reporting. For further information on Nature Research policies, see Authors & Referees and the Editorial Policy Checklist.

Statistical parameters

When statistical analyses are reported, confirm that the following items are present in the relevant location (e.g. figure legend, table legend, main text, or Methods section).

n/a	Confirmed	
☐	☒	The exact sample size (n) for each experimental group/condition, given as a discrete number and unit of measurement
☐	☒	An indication of whether measurements were taken from distinct samples or whether the same sample was measured repeatedly
☐	☒	The statistical test(s) used AND whether they are one- or two-sided<br>Only common tests should be described solely by name; describe more complex techniques in the Methods section.
☐	☒	A description of all covariates tested
☐	☒	A description of any assumptions or corrections, such as tests of normality and adjustment for multiple comparisons
☐	☒	A full description of the statistics including central tendency (e.g. means) or other basic estimates (e.g. regression coefficient) AND variation (e.g. standard deviation) or associated estimates of uncertainty (e.g. confidence intervals)
☐	☒	For null hypothesis testing, the test statistic (e.g. F, t, r) with confidence intervals, effect sizes, degrees of freedom and P value noted<br>Give P values as exact values whenever suitable.
☒	☐	For Bayesian analysis, information on the choice of priors and Markov chain Monte Carlo settings
☐	☒	For hierarchical and complex designs, identification of the appropriate level for tests and full reporting of outcomes
☐	☒	Estimates of effect sizes (e.g. Cohen's d, Pearson's r), indicating how they were calculated
☐	☒	Clearly defined error bars<br>State explicitly what error bars represent (e.g. SD, SE, CI)

Our web collection on statistics for biologists may be useful.

Software and code

Policy information about availability of computer code

Data collection	All images were obtained using Nikon Eclipse Ti microscope with a 60X oil objective and Nikon ND acquisition software.
Data analysis	For imaging, the raw TIFF files (images) obtained were analyzed using custom-written and previously published MATLAB scripts (Senaratne, T. N., Joyce, E. F., Nguyen, S. C. & Wu, C. T. Investigating the Interplay between Sister Chromatid Cohesion and Homolog Pairing in Drosophila Nuclei. PLoS genetics 12, e1006169, doi:10.1371/journal.pgen.1006169 (2016).)<br>For Hi-C data, and WGS data:<br>Data analysis was performed using: seqtk trimfq v.1.2-r94 (https://github.com/lh3/seqtk), bwa mem v.0.7.15, pairtools parse command line tool (https://github.com/mirnylab/pairtools), the cooler package (https://github.com/mirnylab/cooler), the package cooltools insulation (https://github.com/mirnylab/cooltools), peakdet: Peak detection using MATLAB, http://billauer.co.il/peakdet.html), Jupyter Notebooks, matplotlib, numpy, pandas. We automated data analyses in command line interface using GNU Parallel. Details included in methods section. The software used in this study is available at https://github.com/mirnylab/

For manuscripts utilizing custom algorithms or software that are central to the research but not yet described in published literature, software must be made available to editors/reviewers upon request. We strongly encourage code deposition in a community repository (e.g. GitHub). See the Nature Research guidelines for submitting code & software for further information.

Data

Policy information about availability of data

All manuscripts must include a data availability statement. This statement should provide the following information, where applicable:

- Accession codes, unique identifiers, or web links for publicly available datasets
- A list of figures that have associated raw data
- A description of any restrictions on data availability

All raw sequencing data and extracted Hi-C contacts have been deposited in the Gene Expression Omnibus (GEO) repository under accession number GSE121256

Field-specific reporting

Please select the best fit for your research. If you are not sure, read the appropriate sections before making your selection.

☒ Life sciences ☐ Behavioural & social sciences ☐ Ecological, evolutionary & environmental sciences

For a reference copy of the document with all sections, see nature.com/authors/policies/ReportingSummary-flat.pdf

Life sciences study design

All studies must disclose on these points even when the disclosure is negative.

Sample size	We used sample size commonly typical for the type of experiments. For Hi-C experiments, we generated 2 biological replicates for each experimental condition, and for FISH experiments, we counted at least 2-3 replicates with an average of 100 nuclei/replicate.
Data exclusions	For Hi-C analyses, bins with low-coverage were excluded.
Replication	All attempts at data replication were successful.
Randomization	The samples were not randomly allocated into experimental groups. Samples were treated in parallel with internal controls to minimize potential confounding effects.
Blinding	The investigators were not blinded during data collection and analysis.

Reporting for specific materials, systems and methods

Materials & experimental systems

n/a	Involved in the study	
☐	☒	Unique biological materials
☐	☒	Antibodies
☐	☒	Eukaryotic cell lines
☒	☐	Palaeontology
☒	☐	Animals and other organisms
☒	☐	Human research participants

Methods

n/a	Involved in the study	
☒	☐	ChIP-seq
☐	☒	Flow cytometry
☒	☐	MRI-based neuroimaging

Unique biological materials

Policy information about availability of materials

Obtaining unique materials	The cell line generated in this study is a fully phased, clonal, diploid and produced from ~2-14 hr old hybrid embryos (generated from a cross from another publication: DGRP-057 (virgin females) and 439 (males) strains (Bloomington stock numbers 29652 and 29658)) (bioRxiv: Erceg, J. et al. The genome-wide, multi-layered architecture of chromosome pairing in early Drosophila embryos bioRxiv (2018)). The hybrid cell line PatnMat (PnM) is a unique cell line that is publicly available upon request and post publication.

Antibodies

Antibodies used	- phosphohistone H3 (P-H3; rabbit used at 1:100; Abcam (Ab32107))

	-A Cy3-conjugated anti-rabbit secondary antibody (Jackson ImmunoResearch Laboratories (111-025-003))<br>-Rabbit anti-dMef2 antibody, which was a gift from Bruce Paterson<br>-anti-GFP antibody (ab290, 1:1000)<br>-anti-α-tubulin antibody (ab40742; 1:5000)<br>-rabbit secondary antibody conjugated to HRP (at 1:5000:)
Validation	-Anti-P-H3 was used and validated previously (Senaratne, T. N., Joyce, E. F., Nguyen, S. C. & Wu, C. T. Investigating the Interplay between Sister Chromatid Cohesion and Homolog Pairing in Drosophila Nuclei. PLoS genetics 12, e1006169, doi:10.1371/journal.pgen.1006169 (2016))<br>-Anti-dMef2 was used, and validated previously (Lilly, B. et al. Requirement of MADS domain transcription factor D-MEF2 for muscle formation in Drosophila. Science (New York, N.Y.) 267, 688-693 (1995))<br>-Anti-GFP was used, and validated previously in Wu D et al, RanGAP-mediated nucleocytoplasmic transport of Prospero regulates neural stem cell lifespan in Drosophila larval central brain, Aging Cell, (2019).

Eukaryotic cell lines

Policy information about cell lines

Cell line source(s)	The cell line (PnM) was generated for this study from 2-14 hr old embryos.
Authentication	This study validates that the cell line is diploid, fully phased, and produced from divergent lines (hybrid).
Mycoplasma contamination	Cell lines were not tested for mycoplasma contimaination
Commonly misidentified lines<br>(See ICLAC register)	There are no other cell lines or commonly used cell lines used in this study.

Flow Cytometry

Plots

Confirm that:

☒ The axis labels state the marker and fluorochrome used (e.g. CD4-FITC).

☒ The axis scales are clearly visible. Include numbers along axes only for bottom left plot of group (a 'group' is an analysis of identical markers).

☒ All plots are contour plots with outliers or pseudocolor plots.

☒ A numerical value for number of cells or percentage (with statistics) is provided.

Methodology

Sample preparation	Cells were split a day prior and then 1-3 million cells were harvested and fixed with 95% ethanol, washed with 1X PBS and stained using FxCycle PI/RNase staining solution (Life Technologies) for 30 min at room temperature.
Instrument	Cell populations were assayed based on DNA content to determine their cell cycle profile using an LSR II Analyzer
Software	FlowJo VX
Cell population abundance	G0/G1: 63.3%, S: 22.8%, G2/M: 13.9%
Gating strategy	Cells were sorted at 70% confluency, FSC (320), SSC (291)

☒ Tick this box to confirm that a figure exemplifying the gating strategy is provided in the Supplementary Information.
